# Double Proton Transfer in the Dimer of Formic Acid: An Efficient Quantum Mechanical Scheme

**DOI:** 10.3389/fchem.2019.00676

**Published:** 2019-10-23

**Authors:** Hao Liu, Jianwei Cao, Wensheng Bian

**Affiliations:** ^1^Beijing National Laboratory for Molecular Sciences, Institute of Chemistry, Chinese Academy of Sciences, Beijing, China; ^2^School of Chemical Sciences, University of Chinese Academy of Sciences, Beijing, China

**Keywords:** tunneling splitting, proton transfer, quantum dynamics, formic acid dimer, normal coordinates

## Abstract

Double proton transfer plays an important role in biology and chemistry, such as with DNA base pairs, proteins and molecular clusters, and direct information about these processes can be obtained from tunneling splittings. Carboxylic acid dimers are prototypes for multiple proton transfer, of which the formic acid dimer is the simplest one. Here, we present efficient quantum dynamics calculations of ground-state and fundamental excitation tunneling splittings in the formic acid dimer and its deuterium isotopologues. These are achieved with a multidimensional scheme developed by us, in which the saddle-point normal coordinates are chosen, the basis functions are customized for the proton transfer process, and the preconditioned inexact spectral transform method is used to solve the resultant eigenvalue problem. Our computational results are in excellent agreement with the most recent experiments (Zhang et al., 2017; Li et al., 2019).

## 1. Introduction

Proton transfer plays important roles in various chemical and biological processes (Mayer, 2011; Weinberg et al., 2012; Layfield and Hammes-Schiffer, 2014; Salamone and Bietti, 2015). Multiple proton transfer is nearly ubiquitous in living organisms, such as in DNA mutation reactions (Jacquemin et al., 2014) or enzyme catalysis reactions (Klinman and Kohen, 2013). In particular, the hydrogen bond is crucial and omnipresent in many chemical and biological reactions, and in case that more than one hydrogen bond exist, different multiple proton transfer processes along the corresponding hydrogen bonds would appear, either in a concerted or stepwise way. In this field, the double proton transfer systems are of extraordinary importance as they can serve as the template for DNA base pairs (Barnes et al., 2008; Smedarchina et al., 2018). The carboxylic acid dimers are often used as models for multiple proton concerted transfer (Arabi and Matta, 2011; Daly et al., 2011; Evangelisti et al., 2012; Feng et al., 2012; Zhou et al., 2019), of which the formic acid dimer (FAD) is the smallest one. Therefore, the FAD system has long been considered as the prototype for multiple proton transfer studies (Li et al., 2019). Of course, it should be noted that a realistic simulation of the proton transfer processes in the real biological environment would require a more complex model, since such factors as the surrounding water molecules (Cerón-Carrasco et al., 2010; Cerón-Carrasco and Jacquemin, 2015) and the local electric field (Arabi and Matta, 2011) have been shown to play important roles.

**Figure 1:**
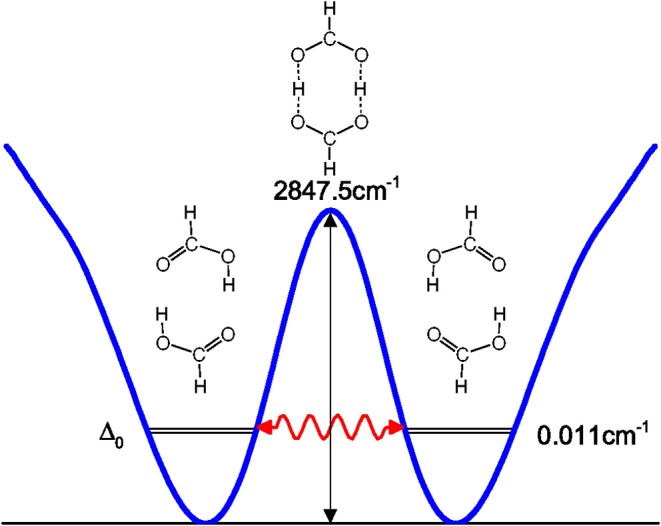
Proton transfer along the isomerization path of formic acid dimer.

Tunneling splittings can provide direct information about dynamics of proton transfer, and it can be detected by high-resolution spectroscopic techniques (Zielke and Suhm, 2007; Daly et al., 2011; Goroya et al., 2014; MacKenzie et al., 2014; Zhang et al., 2017; Li et al., 2019). As shown in Figure 1, hydrogen (protons) in FAD can transfer between oxygen via tunneling, resulting in vibrational energy level splitting. Experimentally, Li et al. (2019) have just performed the most accurate measurement of ground-state tunneling splitting of FAD with microwave spectroscopy, with the splitting value reported as 334.9 MHz (0.01117 cm^−1^). In 2002, Havenith's group (Madeja and Havenith, 2002) first successfully employed high-resolution spectroscopy to measure tunneling splitting in (DCOOH)_2_. More recently, Havenith's group (Ortlieb and Havenith, 2007) and Duan's group (Goroya et al., 2014) measured ground-state tunneling splitting(Δ_0_) of (HCOOH)_2_ as 0.0158 and 0.01649 cm^−1^, respectively. In 2017, Duan's group (Zhang et al., 2017) improved their experimental accuracy, getting an updated Δ_0_ of 0.011367(92) cm^−1^ for (HCOOH)_2_, and they also reported a new experimental Δ_0_ of 0.00113 cm^−1^ for HCOOD-HCOOH. Theoretically, several researchers studied the tunneling splittings in the FAD system using approximate methods, such as instanton theory (Mil'nikov et al., 2005; Smedarchina et al., 2005, 2013; Richardson, 2017) and reduced-dimensionality quantum dynamics (QD) (Luckhaus, 2006, 2010; Barnes et al., 2008; Jain and Sibert, 2015; Qu and Bowman, 2016). In 2016, Qu and Bowman successfully constructed a full-dimensional potential-energy surface (PES) for FAD (Qu and Bowman, 2016), which provides us the basis for further dynamical calculations. Based on this PES, two kinds of dynamical calculations have been reported, which are reduced-dimensional quantum calculations with the multi-mode method (Qu and Bowman, 2016) and semiclassical calculations with the instanton approach by Richardson (Richardson, 2017). However, the agreement of the reported theoretical values for the ground-state tunneling splittings with the most recent experiments (Zhang et al., 2017; Li et al., 2019) is still not satisfactory. The ground-state tunneling splitting for FAD is very small (only 0.01 cm^−1^ or so), which causes problems for some approximate approaches such as diffusion Monte Carlo (Qu and Bowman, 2016), and in some cases the numerical errors may be larger than the splitting value. In addition, although great efforts have been made in full-dimensional exact QD calculations (Wu et al., 2016; Pandey and Poirier, 2019), the full-dimensional exact QD calculations are still prohibitive for the title 10-atom system.

In this work, we present efficient QD calculations of ground-state and fundamental excitation tunneling splittings in FAD using the PES of Bowman's group (Qu and Bowman, 2016), and as for the ground-state tunneling splitting, our calculations yield much better agreement with experiments (Zhang et al., 2017; Li et al., 2019) than previous theoretical calculations. These are achieved with a multidimensional scheme developed by us, in which the saddle-point normal coordinates are chosen and vibrational modes that are strongly coupled to the proton transfer are included. The basis functions are customized for the proton transfer process using the process-oriented basis function customization (PBFC) strategy proposed by us (Ren and Bian, 2015; Wu et al., 2016), and the preconditioned inexact spectral transform (PIST) method (Huang and Carrington, 2000; Poirier and Carrington, 2001, 2002; Ren et al., 2011; Yang et al., 2011) is used to solve the resultant eigenvalue problem. The main idea of our PBFC strategy is to customize basis functions for specific chemical processes or those desired states by optimizing and adjusting the 1D or nD effective potential (EP).

## 2. Methods and Computational Details

### 2.1. Normal Mode Hamiltonian

The exact normal mode Hamiltonian of a non-linear system for total angular momentum *J* = 0 reads (Kamarchik et al., 2009)

(1) $$\begin{array}{l} \hat{H}=-\frac{1}{2}\underset{i=1}{\sum }\frac{\partial^{2}}{\partial Q_{i}^{2}}+\frac{1}{2}\underset{\alpha ,\beta }{\sum }{\hat{\pi }}_{\alpha }\mu_{\alpha \beta }{\hat{\pi }}_{\beta }-\frac{1}{8}\underset{\alpha }{\sum }\mu_{\alpha \alpha }+V\left(\text{Q}\right) \end{array}$$

(2) $$\begin{array}{l} {\hat{\pi }}_{\alpha }=-i\underset{k,l}{\sum }\zeta_{k,l}^{\alpha }Q_{k}\frac{\partial }{\partial Q_{i}} \end{array}$$

where **Q** denotes a collection of the 3*N*−6 normal coordinates, μ_αβ_ is the inverse of the effective moment of inertia tensor, and $\zeta_{k,l}^{\alpha }$ are the Coriolis coupling coefficients. The four terms are the standard kinetic energy operator, the vibrational angular momentum (VAM) term, the so-called “Watson” term, and the potential term in order. As VAM and Watson are inverse with the moment of inertia, we can neglect them in this 10-atom system. Therefore, the expression of multidimensional effective Hamiltonian reads (Ren et al., 2011; Wu, 2016)

(3) $$\hat{H}=-\frac{1}{2}\sum_{i=1}^{M}\frac{\partial^{2}}{\partial Q_{i}^{2}}+V(Q_{1},\ldots ,Q_{M})$$

where *M* is the number of modes included in this calculation. *V*(*Q*_1_, …, *Q*_*M*_) is the EP obtained by customized according the reaction process or simply minimizing the remaining degrees of freedom (DOF). The criterion for choosing normal modes will be discussed later in the article.

For facilitating description of proton tunneling in FAD, the Hamiltonian is represented in the saddle-point normal coordinates as the saddle point has the highest symmetry. Normal mode analysis is performed employing the PES constructed by Bowman's group (Qu and Bowman, 2016). We chose the direction of the saddle point painstakingly to avoid the symmetrical error caused by the numerical problem. The mass-scaled normal modes obtained at the saddle point and global minimum are provided in Table 1, in which the imaginary frequency *Q*_1_ is the reaction coordinate.

**Table 1:** Correspondence between the normal modes of the saddle point (SP) and the global minimum (GM).

**Saddle point**			**Global minimum**				**Expt**.	
**Mode**	**ω/cm^−1^**	**Motion^a^**	**Mode**	**ω/cm^−1^**	**Γ^a^**	**Coeff^b^**	**mode^c^**	**ν/cm^−1^**
1	1355.32i	PT	23′	3232.02	*A*_*g*_	0.90	1	3,570^e^
2	79.80	τ_*R*_	1′	70.08	*A*_*u*_	0.99	16	69.2^c^
3	218.58	β_*R*_	2′	167.10	*A*_*g*_	0.92	9	165^f^
4	225.75	δ_*R*_	3′	170.48	*A*_*u*_	0.99	15	168.47^c^
5	317.20	δ_*R*_	5′	253.96	*B*_*g*_	0.98	12	242^f^
6	513.81	ν_*R*_	4′	209.11	*A*_*g*_	0.92	8	194^f^
7	591.67	β_*R*_	6′	275.43	*B*_*u*_	0.98	24	268^c^
8	744.42	β_*OCO*_	7′	692.87	*A*_*g*_	0.75	7	682^h^
9	813.83	β_*OCO*_	8′	715.98	*B*_*u*_	0.94	23	698^c^
10	1065.35	δ_*CH*_	11′	1084.35	*B*_*g*_	0.96	10	1,060^d^
11	1078.91	δ_*CH*_	12′	1100.26	*A*_*u*_	0.83	13	1033.5^e^
12	1240.46	ν_*OH*_	24′	3326.01	*B*_*u*_	0.92	17	3,084^c^
13	1341.34	δ_*OH*_	9′	956.00	*B*_*g*_	0.89	11	–
14	1394.98	β_*OCH*_	15′	1405.97	*B*_*u*_	0.77	21	1,364^c^
15	1397.27	β_*OCH*_	16′	1408.36	*A*_*g*_	0.83	5	1,375^d^
16	1400.00	δ_*OH*_	10′	969.97	*A*_*u*_	0.82	14	922^c^
17	1403.61	ν_*CO*_(+)	14′	1258.45	*B*_*u*_	0.71	22	1,218^c^
18	1408.33	ν_*CO*_(+)	13′	1255.42	*A*_*g*_	0.70	6	1,214^d^
19	1603.78	β_*OH*_	17′	1447.95	*B*_*u*_	0.85	20	1,454^c^
20	1691.31	β_*OH*_	18′	1480.88	*A*_*g*_	0.64	4	1,379^g^
21	1742.84	ν_*CO*_(−)	20′	1779.77	*B*_*u*_	0.88	19	1,746^c^
22	1748.51	ν_*CO*_(−)	19′	1714.78	*A*_*g*_	0.78	3	1,670^d^
23	3101.02	ν_*CH*_	21′	3095.24	*A*_*g*_	1.00	2	2943.8^e^
24	3106.48	ν_*CH*_	22′	3096.71	*B*_*u*_	0.99	18	2938.5^c^

a *PT means the proton transfer mode, ν is stretch, β is in-plane bend, δ is out-of-plane bend, τ is torsion, R is intermolecular, ± is symmetric or antisymmetric. Γ is the irreps of the C_2h_ point group used to label the vibrational modes*.

b *coeff gives the corresponding dot product of normal mode vectors of the SP and the GM configuration*.

c *Georges et al. (2004)*.

d *Bertie et al. (1986)*.

e *Baskakov et al. (2006)*.

f *Zielke and Suhm (2007)*.

g *Luo et al. (2017)*.

h *Xue and Suhm (2009)*.

### 2.2. Basis Function Representation

**Figure 2:**
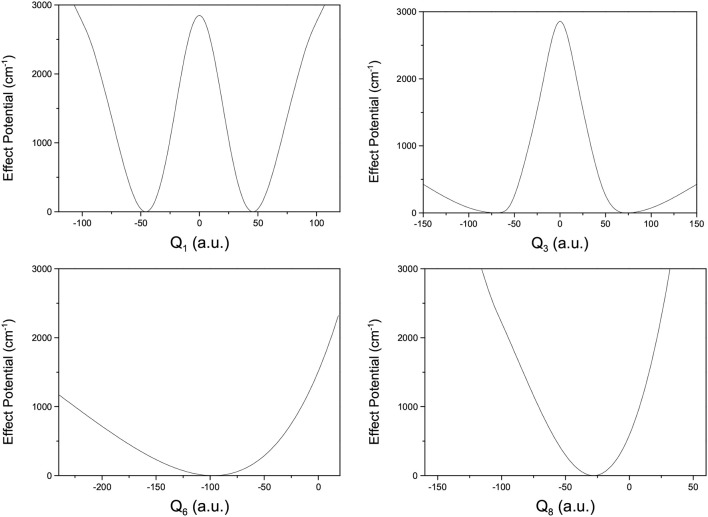
1D effective potentials for *Q*_*i*_(*i* = 1, 6, 3, 8) in formic acid dimer, where *Q*_*i*_ is in the mass-rescaled unit.

**Figure 3:**
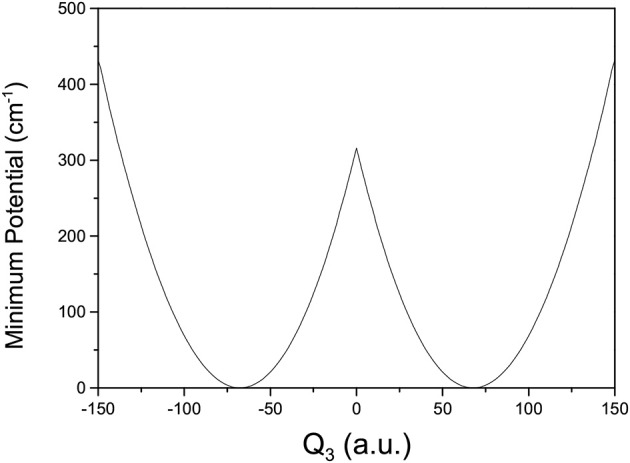
1D minimum potentials for *Q*_3_ in formic acid dimer, where *Q*_3_ is in the mass-rescaled unit.

The wave function is expanded by the direct product of 1D discrete variable representation (DVR) basis functions

(4) $$\begin{array}{l} \Psi =\sum_{i_{1}=1}^{N_{1}}\ldots \sum_{i_{M}=1}^{N_{M}}c_{i_{1}\ldots i_{M}}\overset{M}{\underset{j=1}{\Pi }}\pi_{i_{j}}(Q_{j}) \end{array}$$

where π_*i*_*j*__(*Q*_*j*_) is the 1D DVR basis function for *Q*_*j*_ with basis size of *N*_*j*_. The 1D DVR basis functions are obtained by a designed 1D effective Hamiltonian with a unitary transformation from the truncated eigenfunctions

(5) $$\begin{array}{l} {\hat{H}}_{Q_{j}}=-\frac{1}{2}\frac{\partial^{2}}{\partial Q_{j}^{2}}+V\left(Q_{j}\right) \end{array}$$

where *V*(*Q*_*j*_) is the 1D EP (Li et al., 2011; Ren et al., 2011; Zhang et al., 2012). The two protons in FAD transfer between the two equivalent wells results in ground-state tunneling splitting (Figure 1), and in normal coordinates at the saddle point, *Q*_1_ is identified as the proton transfer reaction coordinate as shown in Table 1. The PBFC strategy is used to customize the 1D EP for the proton tunneling process attracting our interest, and the four 1D EPs used in this work are shown in Figure 2. In particular, the 1D EP for *Q*_3_ is obtained by smoothly connecting three parts: the central part is yielded by following the steepest descending path starting from the saddle point, whereas the parts on the two sides are produced by minimizing all the remaining DOF. It is clear that the obtained 1D EP for *Q*_3_ is proton-transfer-process oriented, which includes the reactant and product equilibrium geometries and the transition-state geometry. If the EP for *Q*_3_ is generated by minimizing all the remaining DOF, a segmented point or cusp at *Q*_3_ = 0 will appear (see Figure 3), and on the two sides of the cusp the relaxed coordinate *Q*_1_ has the opposite sign, indicating that it just describes the well regions but omits the barrier region. In the following we will show that the coupling between *Q*_1_ and *Q*_3_ is anti-symmetric, and generally speaking, whenever this kind of strong anti-symmetric coupling is encountered, the above problem would appear. In addition, the 1D EP for the mode *Q*_1_ can be obtained in a similar way to that for *Q*_3_. The 1D EPs for the modes *Q*_6_ and *Q*_8_ are generated from the full-dimensional PES by minimizing the potential with all the remaining DOF, respectively, which is in accordance with the spirit of the PBFC strategy, since the minimum potential is energetically favored in the process of proton transfer. It should be noted that the minimal potentials have been shown to give rise to nearly optimal effective Hamiltonians using the phase space optimizing (PSO) theory (Poirier and Light, 1999, 2001; Poirier, 2001; Bian and Poirier, 2003).

Figure 2 shows that the 1D EP for *Q*_1_ has a double-well structure, as does that for *Q*_3_, indicating that the *Q*_1_ and *Q*_3_ modes may be the most important in the study of tunneling splitting. Furthermore, the elements in the Hamiltonian matrix we use for this work are the following,

(6) $$\begin{array}{l} {\left[\hat{H}\right]}_{i_{1}i_{2}\ldots i_{M},\;i_{1}^{\prime }i_{2}^{\prime }\ldots i_{M}^{\prime }}=T_{i_{1}i_{1}^{\prime }}^{Q_{1}}\delta_{i_{2}i_{2}^{\prime }}\ldots \delta_{i_{M}i_{M}^{\prime }}+\delta_{i_{1}i_{1}^{\prime }}T_{i_{2}i_{2}^{\prime }}^{Q_{2}}\ldots \delta_{i_{M}i_{M}^{\prime }}\\ \;+\cdots +\delta_{i_{1}i_{1}^{\prime }}\delta_{i_{2}i_{2}^{\prime }}\ldots T_{i_{M}i_{M}^{\prime }}^{Q_{M}}\\ \;+V\left(Q_{1\;i_{1}},Q_{2\;i_{2}},\ldots ,Q_{M\;i_{M}}\right)\delta_{i_{1}i_{1}^{\prime }}\delta_{i_{2}i_{2}^{\prime }}\ldots \delta_{i_{M}i_{M}^{\prime }} \end{array}$$

where $T^{Q_{j}}$ refers to the kinetic energy matrix in DVRs for *Q*_*j*_, *M* = (1, 2, 3, 4).

### 2.3. Hamiltonian Martix Solution

The essence of the PIST method is to transform the original **H**^−1^ into matrix (**H**−*E***I**)^−1^ before the Lanczos algorithm is applied, such that states close to energy *E* converge first and fast to reduce the number of Lanczos iterations needed. In each Lanczos iteration, the matrix-vector multiplication ${\text{x}}_{i+1}={\left(\text{H}-E\text{I}\right)}^{-1}{\text{x}}_{i}$ is equivalent to the linear equations (**H** − *E***I**)**x**_*i*+1_ = **x**_*i*_, which are solved with the quasi-minimal residual (QMR) algorithm. Wyatt preconditioner (Wyatt, 1995a,b), **P**, is employed to improve the efficiency of the QMR iterative convergence by transforming the linear equations into ${\text{P}}^{-1}\left(\text{H}-E\text{I}\right){\text{x}}_{i+1}={\text{P}}^{-1}{\text{x}}_{i}$, as the matrix **P**^−1^(**H** − *E***I**) is more close to a diagonal matrix. QMR convergence criteria can be loosened to a certain extent, as the exact eigenvalues and eigenvectors are given by the Lanczos step. However, loosening it too much will highly increase the number of steps for Lanczos iteration, which is much slower than QMR iteration. The PIST method has also been employed in other applications (Bian and Poirier, 2004; Li and Bian, 2008; Brandon and Poirier, 2014; Petty and Poirier, 2014).

## 3. Results and Discussion

**Figure 4:**
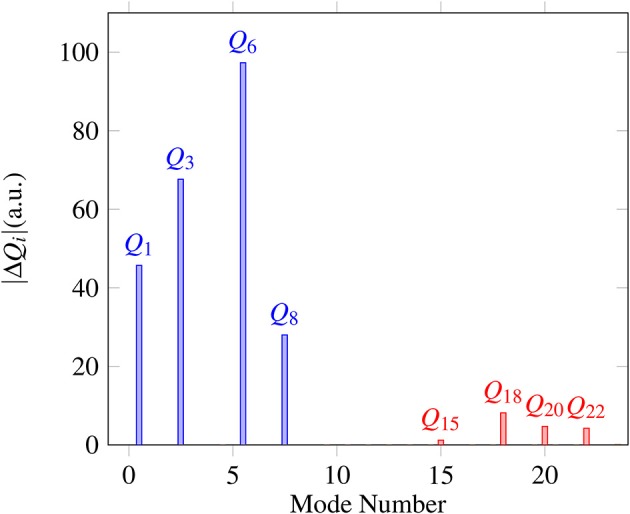
Magnitudes of the displacement for 24 normal coordinates from the saddle point to the global minimum, where |Δ*Q*_*i*_|(*i* = 1, 2, …, 24) is in the mass-rescaled unit.

**Figure 5:**
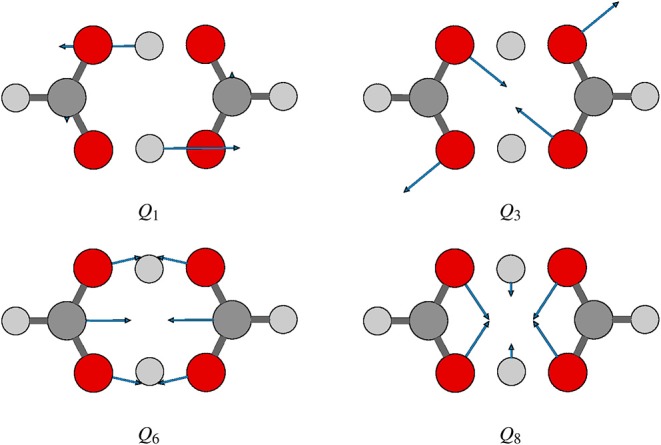
Physical descriptions of most important saddle-point normal modes.

**Figure 6:**
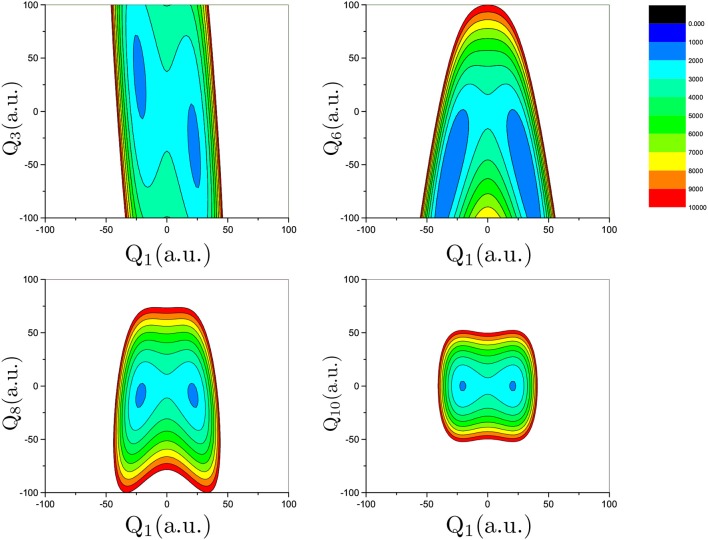
Contour plot of the PES cut along *Q*_*i*_ (*i* = 3, 6, 8, 10) and *Q*_1_ by fixing the remaining modes at zero. The potential energy is in cm^−1^ and *Q*_*i*_ is in the mass-rescaled unit.

Proton transfer in FAD is a multidimensional process. In order to identify important normal coordinates related to the proton transfer of FAD and incorporate them into the current multidimensional research, we first inspect the magnitude of the displacement [|Δ*Q*_*i*_| (*i* = 1, …, 24)] for each normal coordinate from the saddle point to the global minimum. As shown in Figure 4, the |Δ*Q*_*i*_|s of four modes, modes 1, 3, 6, and 8 (Figure 5), are substantially larger than those of the other modes. The contour plots of the PES cut along *Q*_*i*_(*i* = 3, 6, 8, 10) and *Q*_1_ are shown in Figure 6, as seen, the coupling between modes 3 and 1 and that between modes 6 and 1 are extremely strong, while the coupling between modes 10 and 1 is very small. The contour plot of the PES cut along *Q*_22_ and *Q*_1_ is similar to that along *Q*_3_ and *Q*_1_, but the coupling between modes 22 and 1 is much smaller. That confirms the importance of *Q*_6_, *Q*_3_, *Q*_8_, which are used as the main mode in the previous work (Barnes et al., 2008; Jain and Sibert, 2015; Qu and Bowman, 2016; Richardson, 2017). The only replacement may be using *Q*_22_ instead of *Q*_8_ (Matanovi et al., 2008).

As what written above, for the calculation of ground-state splitting (Δ_0_), *Q*_6_, *Q*_3_, and *Q*_8_ are extracted from the 4D (*Q*_1_, *Q*_6_, *Q*_3_, *Q*_8_) model. Converged ground-state splittings are obtained with the basis set of (*N*_*Q*_1__ = 32, *N*_*Q*_6__ = 13, *N*_*Q*_3__ = 13, *N*_*Q*_8__ = 11) which is denoted as (32, 13, 13, 11) for simplicity. In analyzing the EPs, we find that the coordinate with |Δ*Q*_*i*_| ≈ 0 (Figure 4) leaves from 0 only in the region where the FAD breaks into two monomers. Thus, when making multidimensional EPs, we relax the modes shown in Figure 4 and keep the others near 0 to make our calculations focus on the process of isomerization.

Because the theoretical splittings are computed with the saddle point coordinates whereas the experiments are measured as a property of the global minimum, we establish the corresponding relations between the saddle-point modes and the global minimum ones to compare our splittings of each mode with experiment. The relations of the different mode numbers are shown in Table 1.

The calculated ground-state tunneling splitting results for (HCOOH)_2_ are listed in Table 2. As shown, the present 3-4D results agree well with the experimental measurements, which are superior to the previous 3-4D results (Qu and Bowman, 2016; Richardson, 2017). The good performance of the present calculations may be attributed to two reasons. First, the potential energies used in the calculations of Sibert's group (Barnes et al., 2008; Jain and Sibert, 2015) and Došlić's group (Matanovi et al., 2008) are only at the B3LYP level and not accurate enough, and similar problem is also found in the 7D calculation of Luckhaus (Luckhaus, 2010) which reported a value of 0.008 cm^−1^. Second, although we use the same PES as that used in Bowman's calculations, we treat the symmetry problem in calculations with great care and obtain more reliable results. We find that the symmetry of PES breaks while converting the coordinate from Cartesian coordinate to normal coordinate. When the two wells are not in symmetry with each other, the wave-functions of the doublets that one state splits into will be independently bonded in one well instead of spreading in both wells, so the two doublets are broken into two states. Although this symmetry problem is not obvious in 1D calculation, it does affect the 2-4D results.

**Table 2:** Ground-state tunneling splitting for (HCOOH)_2_, energies in cm^−1^.

	**This work**	**Bowman^a^**	**Richardson^b^**	**Sibert (2008)^c^**	**Sibert (2015)^d^**	**Došlić^e^**
*Q*_1_	0.4404	0.44	0.47			
*Q*_1_, *Q*_6_	0.05865	0.16	0.17			
*Q*_1_, *Q*_3_	0.03586					
*Q*_1_, *Q*_6_, *Q*_3_	0.01136	0.032	0.037	0.0063	0.0017	0.163
*Q*_1_, *Q*_6_, *Q*_3_, *Q*_8_	0.01027	0.037	0.047			
Expt.	0.01117^f^					
	0.011367(92)^g^					
	0.0158(4)^h^					

a *Qu and Bowman (2016)*.

b *Richardson (2017)*.

c *Use B3LYP/6-31+G(d) for the potential, see (Barnes et al., 2008)*.

d *Jain and Sibert (2015)*.

e *Use B3LYP/6-311++G(3df, 3pd) for the potential, see (Matanovi et al., 2008)*.

f *Li et al. (2019)*.

g *Zhang et al. (2017)*.

h *Ortlieb and Havenith (2007)*.

**Figure 7:**
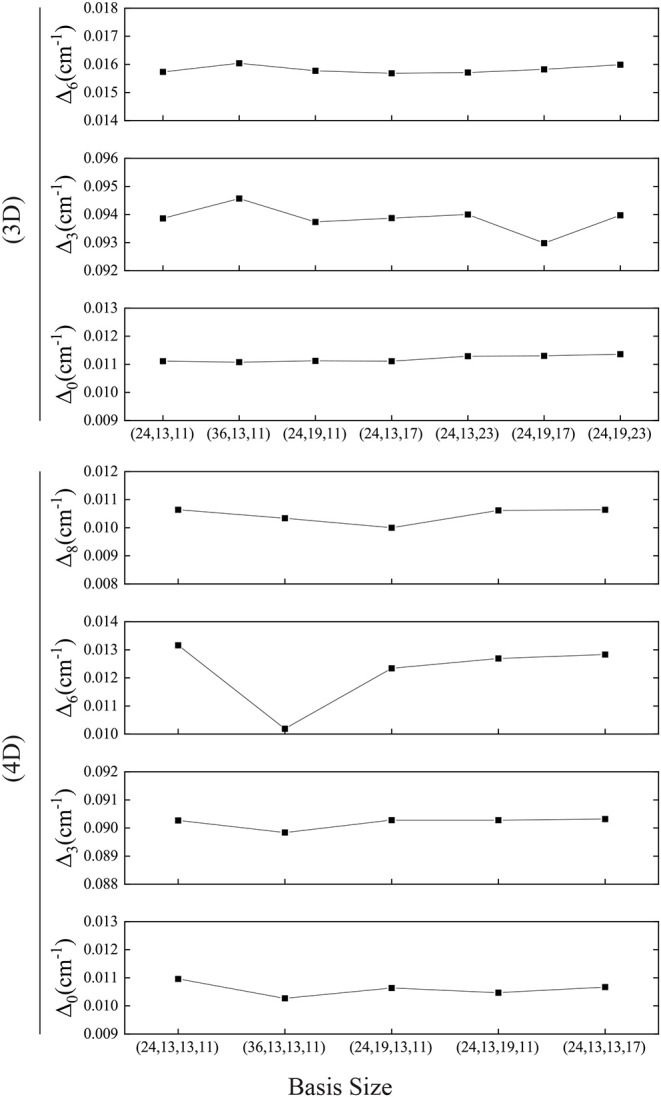
Convergence of the tunneling splittings in 3D or 4D calculations. Basis size is (*Q*_1_, *Q*_6_, *Q*_3_) or (*Q*_1_, *Q*_6_, *Q*_3_, *Q*_8_).

We find that the computer's numerical precision does affect the transformation from the Cartesian coordinates to the normal coordinates, leading to errors, which needs careful treatment to ensure that the zero elements in the coordinate transfer matrix are as expected and the PES in normal coordinates is symmetric. The 4D result is in very good agreement with the experiments (Zhang et al., 2017; Li et al., 2019), and the Δ_0_ in different basis size varies by around 0.0005 cm^−1^ (see Figure 7) which is much smaller than that in Bowman's calculation of 0.003 cm^−1^. In addition, the present computational scheme is efficient. For instance, the calculation with the 4D basis size of (24, 13, 13, 11) takes only 100 s for the PIST part on our workstation with Intel Xeon E5645@2.4GHz, and the most time-consuming part for constructing multidimensional EPs has been parallelized.

We also use the same scheme to calculate the ground-state tunneling splitting of various deuterium isotopologues, and the results for DCOOH-HCOOH (DCOOH)_2_, HCOOD-HCOOH and (HCOOD)_2_ are presented in Table 3. As can be seen, the ground-state tunneling splittings of the four deuterium isotopologues are smaller than that of (HCOOH)_2_, meaning that substituting the hydrogen atoms with the deuterium atom would slow down the tunneling. In particular, for the 4D results, the calculated splitting for DCOOH-HCOOH and HCOOD-HCOOH are 0.00988 and 0.00123 cm^−1^, respectively, which is in very good agreement with the experimental values of 0.01106 cm^−1^ (Li et al., 2019) and 0.00113 cm^−1^ (Zhang et al., 2017). As for (DCOOH)_2_, the present calculated ratio of the tunneling splitting for (HCOOH)_2_/(DCOOH)_2_ of 1.25 is in excellent agreement with the experimental value of 1.21 (Ortlieb and Havenith, 2007). The calculated tunneling splitting of (HCOOD)_2_ is 0.000284 cm^−1^, which is consistent with the reported theoretical results of 0.00022 cm^−1^ (Smedarchina et al., 2005) and 0.00021 cm^−1^ (Richardson, 2017) based on the instanton method; unfortunately, there has been no available experimental data, and further experimental studies are desired.

**Table 3:** Ground-state tunneling splitting for the deuterium isotopologues, energies in cm^−1^.

	**DCOOH-HCOOH**	**DCOOH-DCOOH**		**HCOOD-HCOOH**	**HCOOD-HCOOD**	
	**This work**	**This work**	**Bowman^a^**	**This work**	**This work**	**Richardson^b^**
*Q*_1_	0.427	0.414	0.41	0.0956	0.0190	0.017
*Q*_1_, *Q*_6_	0.0580	0.0537	0.15	0.0106	0.00182	0.0043
*Q*_1_, *Q*_3_	0.0327	0.0342		0.00724	0.00122	
*Q*_1_, *Q*_6_, *Q*_3_	0.0107	0.00871	0.028	0.00141	0.000286	
*Q*_1_, *Q*_6_, *Q*_3_, *Q*_8_	0.00988	0.00767		0.00123	0.000284	
Expt.	0.01106^c^			0.00113^d^	<0.00067^d^	

a *Qu and Bowman (2016)*.

b *Quantum dynamics calculations, see (Richardson, 2017)*.

c *Li et al. (2019)*.

d *Zhang et al. (2017)*.

**Figure 8:**
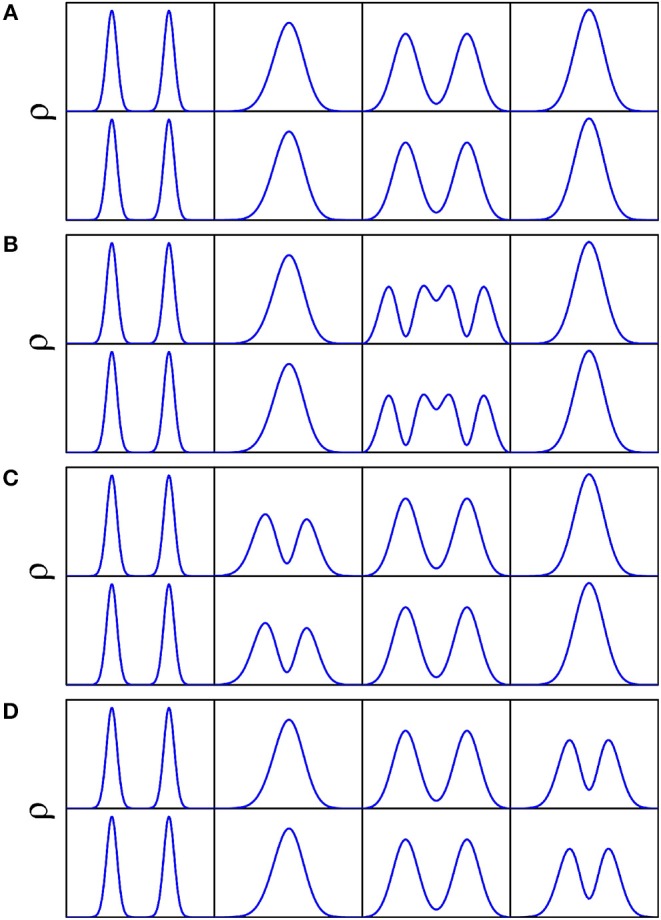
Wave function probability density for the ground state **(A)**, fundamentals Q_3_ **(B)**, Q_6_ **(C)**, and Q_8_ **(D)** against each coordinate with the other coordinates integrated over in the 4D (*Q*_1_, *Q*_6_, *Q*_3_, *Q*_8_) calculation. Top and bottom panels of **(A–D)** correspond to lower and upper doublets of respective tunneling splittings.

The doublets of splittings are assigned according to the nodal structure of wave function probability density against each coordinate, with the other coordinates integrated over. As illustrated in Figure 8 concerning a 4D (*Q*_1_, *Q*_6_, *Q*_3_, *Q*_8_) calculation, Figures 8A–D is the wave function probability density curve of the energy doublets of splittings for the ground state, fundamentals (*Q*_6_, *Q*_3_, *Q*_8_), respectively. There are two potential wells separated by a barrier along *Q*_3_ (Figure 2); considering that the ZPE of *Q*_3_ is about 90 cm^−1^, the wave-functions of *Q*_3_ are divided into the two wells. The number of nodes in wave function of vibrational state of *Q*_3_(ν = *n*) is 2*n*+1, whereas this number of single-well modes like *Q*_3_ or *Q*_8_ is *n*.

One can notice that in Table 4, the effect of the vibrational excitation in mode *Q*_3_ is significantly larger than that in mode *Q*_6_ and *Q*_8_, though the *Q*_6_ has larger displacement and higher frequency. This selective can be found in many systems such as malonaldehyde (Wu et al., 2016). The reason may be that in the tunneling dynamic, the reaction path does not go through the saddle point. For the two global minimums of FAD, *Q*_6_ and *Q*_8_ are nearly the same, while *Q*_3_ has a significant change. Which means that when the protons transfer below the barrier the other atoms will have movement along the mode *Q*_3_ but not along *Q*_6_ and *Q*_8_.

**Table 4:** Ratio of splitting for mode-specific fundamental excitation, relative to the ground state.

	**Frequency**	**Δ**_******i******_/**Δ**_****0****_						
**Mode**	**ω_i_(cm^−1^)^a^**	**this work** **(3D)**	**This work** **(4D)**	**Sibert^b^** **(2008)**	**Sibert^c^** **(2015)**	**Luckhaus^d^**	**Došlić^e^**	**Nakamura^f^**
*Q*_3_	172.66	8.23	8.48	10	11	9	8.95	11
*Q*_6_	206.28	1.40	0.96	1.1	4.7	1	0.56	0.74
*Q*_8_	672.79		0.97			1		1.35

a *Results from this work (4D)*.

b *Barnes et al. (2008)*.

c *Jain and Sibert (2015)*.

d *Luckhaus (2010)*.

e *Matanovi et al. (2008)*.

f *Results in (DCOOH)_2_, see Mil'nikov et al. (2005)*.

In addition, we perform a series of 2D calculations for (*Q*_1_, *Q*_3_) with a basis size up to (48,31); the results are also listed in Table 2. The smaller Δ_0_ indicates that *Q*_3_ does play the second important role in the tunneling; however, being 3 times higher than Δ_0_ in 3D mode shows that *Q*_6_ still shows significant influence to the calculation. For both the 2D cases, we also checked the ratios of Δ_6_/Δ_0_ and Δ_3_/Δ_0_. The results are Δ_6_/Δ_0_ ≈ 4.5, Δ_3_/Δ_0_ ≈ 7.0. Our testing calculation using mode (*Q*_1_, *Q*_3_, *Q*_8_) with a basis size of (24,13,11) gives the result Δ_6_/Δ_0_ ≈ 4.4, far from 1.0, which shows that ignoring *Q*_3_ will affect both the ground state and fundamental excitation tunneling splitting and also reconfirms that *Q*_3_ plays a more important role than *Q*_6_ in the tunneling splitting of FAD.

## 4. Summary

Using a multidimensional scheme developed by us, we achieve much better agreement with experiments than those reported in previous theoretical calculations for the ground-state tunneling splitting in FAD. The obtained ground-state tunneling splitting of 0.010 cm^−1^ is in excellent agreement with the most recent experimental values of 0.011 cm^−1^. This is achieved with a 4-dimensional PBFC-PIST theoretical scheme, in which the saddle-point normal coordinates are chosen, the basis functions are customized for the proton transfer process, and the PIST method is used to solve the resultant eigenvalue problem. Our scheme is also used to study the ground-state tunneling splittings of various deuterium isotopologues of FAD, and the obtained results are in very good agreement with experiment.

The roles of various vibrational modes in the process of proton transfer are also studied, and our analysis and calculations indicate that the *Q*_3_ and *Q*_6_ are strongly coupled to the proton transfer process, whereas *Q*_3_ plays a more important role than *Q*_6_ in the tunneling dynamics. The present work demonstrates the feasibility of our multidimensional PBFC-PIST scheme, which may be extended to the study of multiple proton transfer dynamics in even larger molecular systems or using more complex models, although in the latter case further refinements are required to take into account such factors as the solvent effects by including several explicit water molecules into the model (Cerón-Carrasco et al., 2010).

## Data Availability Statement

All datasets generated for this study are available within the article and from the corresponding author on request.

## Author Contributions

HL carried out the QD calculations. HL, JC, and WB analyzed the data, interpreted the results, developed the theoretical scheme, and wrote the paper. WB supervised the research.

### Conflict of Interest

The authors declare that the research was conducted in the absence of any commercial or financial relationships that could be construed as a potential conflict of interest.
